# Recent Progress for the Techniques of MRI-Guided Breast Interventions and their applications on Surgical Strategy

**DOI:** 10.7150/jca.46329

**Published:** 2020-05-20

**Authors:** Peng Gao, Xiangyi Kong, Ying Song, Yan Song, Yi Fang, Han Ouyang, Jing Wang

**Affiliations:** 1 Department of Breast Surgical Oncology, National Cancer Center/National Clinical Research Center for Cancer/Cancer Hospital, Chinese Academy of Medical Sciences and Peking Union Medical College, Beijing, 100021, China.; 2 Department of Diagnostic Radiology, National Cancer Center/National Clinical Research Center for Cancer/Cancer Hospital, Chinese Academy of Medical Sciences and Peking Union Medical College, Beijing, 100021, China.; 3 Department of Pathology, National Cancer Center/National Clinical Research Center for Cancer/Cancer Hospital, Chinese Academy of Medical Sciences and Peking Union Medical College, Beijing, 100021, China.

**Keywords:** Breast cancer, MRI-guided, Breast surgery, Breast biopsy, Preoperative needle localization

## Abstract

With a high sensitivity of breast lesions, MRI can detect suspicious lesions which are occult in traditional breast examination equipment. However, the lower and variable specificity of MRI makes the MRI-guided intervention, including biopsies and localizations, necessary before surgery, especially for patients who need the treatment of breast-conserving surgery (BCS). MRI techniques and patient preparation should be first carefully considered before the intervention to avoid lengthening the procedure time and compromising targeting accuracy. Doctors and radiologists need to reconfirm the target of the lesion and be very familiar with the process approach and equipment techniques involving the computer-aided diagnosis (CAD) tools and the biopsy system and follow a correct way. The basic steps of MRI-guided biopsy and localization are nearly the same regardless of the vendor or platform, and this article systematically introduces detailed methods and techniques of MRI-guided intervention. The two interventions both face different challenging situations during procedures with solutions given in the article. Post-operative statistics show that the complications of MRI-guided intervention are infrequent and mild, and MRI-guided biopsy provides the pathological information for the subsequent surgical decisions and MRI-guided localization fully prepared for follow-up surgical biopsy. New techniques for MRI-guided intervention are also elaborated in the article, which leads to future development. In a word, MRI-guided intervention is a safe, accurate, and effective technique with a low complication rate and successful MRI-guided intervention is truly teamwork with efforts from patients to surgeons, radiologists, MRI technologists, and nurses.

## Introduction

Breast cancer ranks first among all female tumors, and it tends to be diagnosed at a younger age than other common cancers, with a great threat to women's health and safety 1. The early diagnosis and surgical treatment of breast cancer are of vital importance, especially for young women with the treatment of breast-conserving surgery (BCS, also known as partial mastectomy or lumpectomy). Magnetic resonance imaging (MRI), as an integral tool that is used in combination with mammography and ultrasound, has a sensitivity ranging from 86% to 100% in the detection of breast lesions 2-5. MRI remains the most powerful tool for detecting clinically suspected lesions which are mammographically and sonographically occult. However, the specificity of MRI is between 37% and 97% 6-9. The lower and variable specificity is primarily due to considerable overlap in the appearance of benign and malignant lesions. Thus, breast MRI is often performed as a preoperative examination for suspected lesions that can't be seen with mammography and ultrasound to assess the disease extent. Moreover, MRI-guided localization and biopsy of suspicious lesions are often needed before the surgery, particularly for the young women who deserve to receive BCS. Besides, MRI-guided intervention is a valid technique for the diagnosis of suspicious non-palpable lesions (NPLs) and small masses, which is easy to cause misdiagnosis and delay the treatment of the diseases. These MRI-guided interventions, which are safe, accurate, and effective with a low complication rate that includes infection, hematoma information, and pneumothorax, have transformed the practice of breast imaging 10-14.

Since the 1990s, MRI has been used for breast cancer screening and staging. In 2010, the American College of Radiology (ACR) required facilities to have the equipment to have MRI-guided interventions or have an agreement with the referral center to do so. Successful MRI images analysis for breast tumors is a crucial component for breast surgery 2,15,16. This article reviews the indications and applications of MRI in breast tumors; Reviews the fundamental approaches and necessary steps for performing MRI-guided biopsy and localization in breast tumors, discuss technical challenges in difficult situations with strategies and explores new techniques used in MRI-guided interventions.

## Brief introductions of MRI-guided detection and therapeutic methods in human breast

**Figure 1:**
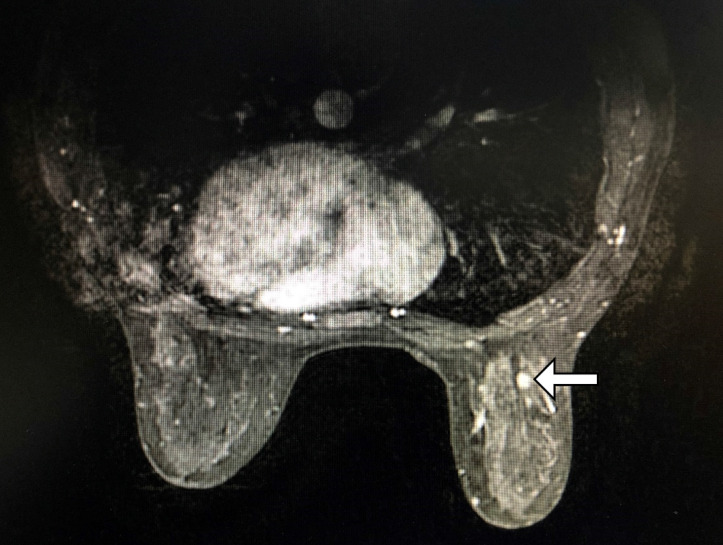
Examples of MRI-detected single right breast lesion of a 47-year-old woman (white horizontal arrow), which is invisible on the corresponding ultrasound and mammography, and the radiologist's report shows BI-RADS 4. This lesion can't be seen on ultrasound after re-identification and then MRI-guided localization is performed with a wire. Pathology after surgery revealed medium nuclear grade ductal carcinoma *in-situ*.

Apart from mammography and ultrasound, women with the most significant risks for developing breast cancer are recommended to have an MRI examination. MRI can give significant additional advice that single or multicentric diseases may not be visible or be detected with traditional breast lesion screening equipment, with a precise determination of the extent of the disease (**Figure 1**). A retrospective review showed that routine pretreatment MRI was not associated with delay in care, with initial surgery occurring in approximately the same interval after diagnosis, and margin-negative surgery actually being accomplished more quickly in patients receiving MRI 17.

What's more, breast MRI has the advantage to detect lesions for high-risk patients. Breast MRI is considered in patients with a lifetime risk of 15-20%, including patients with BRCA1 or BRCA2 mutation, a first-degree relative with a BRCA1 or BRCA2 mutation but who are themselves untested. A personal history of breast or ovarian cancer, and women with a history of high-risk breast lesions such as lobular neoplasia or atypical ductal hyperplasia; patients with a new diagnosis of breast cancer, a known tumor undergoing neoadjuvant chemotherapy, and metastatic lymphadenopathy with an unknown primary cancer are also recommended 18-20.

### Techniques of MRI in Breast

It is better to perform the breast MRI between Day 3 and Day 14 in a typical 28-day menstrual cycle to limit the breast background parenchymal enhancement (BPE) which can render dynamic breast MRI examinations challenging to interpret, due to false-positive findings 21,22. The diagnostic breast MRI is typically performed at either 1.5T or 3T magnetic field strength, and it is generally performed with patients in the prone position using a dedicated breast array coil which can reduce the distortion from respiratory variation when obtaining sequences. An endovenous injection of a paramagnetic contrast agent (such as gadolinium) is usually needed for both morphologic and kinetic features study 23. Although the exact complete diagnostic protocol for breast MRI varies from different institutions, it is often composed of the following sequences: T2 fat-suppressed, pre-contrast T1 no-fat-suppressed, pre-contrast T1 fat-suppressed, and then several postcontrast T1 fat-suppressed images. These examinations take about 40 minutes to complete and vary with the number of sequences acquired 20. Advanced imaging techniques such as diffusion-weighted imaging (DWI) and MR spectroscopy provide complementary information.

Findings are read independently by different breast radiologists with years of experience in breast imaging. They assess the lesions' type (mass, non-mass enhancement, or focus), size, position, shape (circumscribed or irregular), marginal characteristics, and enhancement kinetics. Other characteristics also have to be recorded if not previously logged in the original report. If disagreements are among radiologists with the original report, they need to review the study to reach consensus. All MRI imaging studies are read according to the Breast Imaging Reporting and Data System (BI-RADS) and MRI directional ultrasound is recommended for suspected enhanced lesions (BI-RADS 4 or 5). Final patient treatment is based on an additional conjoint reading of results of all imaging studies 24-26.

### Challenging Clinical Situations

The prone position can help to move the breasts away from the chest wall and minimize respiratory and cardiac motion effects 27. However, the prone position acquires the breasts to be pendant into the coils or fixation plates from immobilization, which is different from the position and situation in the operating room (OR), especially when the surgeon is going to perform BCS. The supine position of patients remains the breast in its native configuration, so the difference between two positions may influence the surgeon when he uses the breast MRI for image guidance of BCS, and the surgeon has to mentally correct for the differences in orientation. In order to overcome this limitation, breast MRI in the supine position has been proposed 14. In one study, patients lie flat on the MR bed with a fixture holding the imaging coil above breasts 28. In this way, the position is more similar to the OR without any constrain or compression, which falls into its native configuration. Besides, the arms of patients have to be parallel to the body while the arms are placed nearly perpendicular to body axes during surgery, which is also a question because the positional change of the arm might cause a non-rigid-body deformation of the adjacent breast 11,29.

## Main technical issues of MRI-guided detection and therapeutic methods in human breast

The breast MRI has been used clinically increasingly over the past decades for its high sensitivity and ability to detect clinically mammographically, and sonographically occult breast cancers. However, due to the lower and variable specificity of MRI, normal breast tissue and other benign lesions can also be enhanced in MRI. So suspicious lesions screened in MRI should be made to re-identify the lesion on mammography or ultrasound to determine whether the lesion can be found again. If the lesion can be re-identified, radiologists firstly choose to perform imaging-guided localization or biopsy with ultrasound and mammography rather than MRI due to the long duration and higher cost, and if a lesion is not clearly identifiable on US or mammography, MRI is used for guidance. Thus, the need for MRI-guided procedures is relatively limited in many institutions 30-33.

But the presumed correlate depicted on the ultrasound image does not truly represent the lesion noted on MRI 34. Amie Y. Lee et al. 35 found that the presumed sonographic correlate was not the true correlate in 26% of 38 cases and that subsequent MRI-guided biopsy of the actual lesion detected on MRI revealed one malignancy in discordant cases. These related researches prove the necessity of the MRI-guided intervention.

### Preparations before MRI-guided Intervention

Before the MRI-guided biopsy or localization, surgery and procedure; radiologists should review clinical information, standard breast imaging, and contrast-enhanced bilateral MRI to establish the quadrant, clock position, depth, distance from nipple, breast density, and surrounding breast tissue landmarks. Other factors which may be assessed include determination of best approach, communication with technologists, optimization of patient positioning, preprocedural checklist confirmation, and accurate targeting of the lesion, which are also critical for maximizing the chances of a safe and successful intervention 2,36.

**Figure 2:**
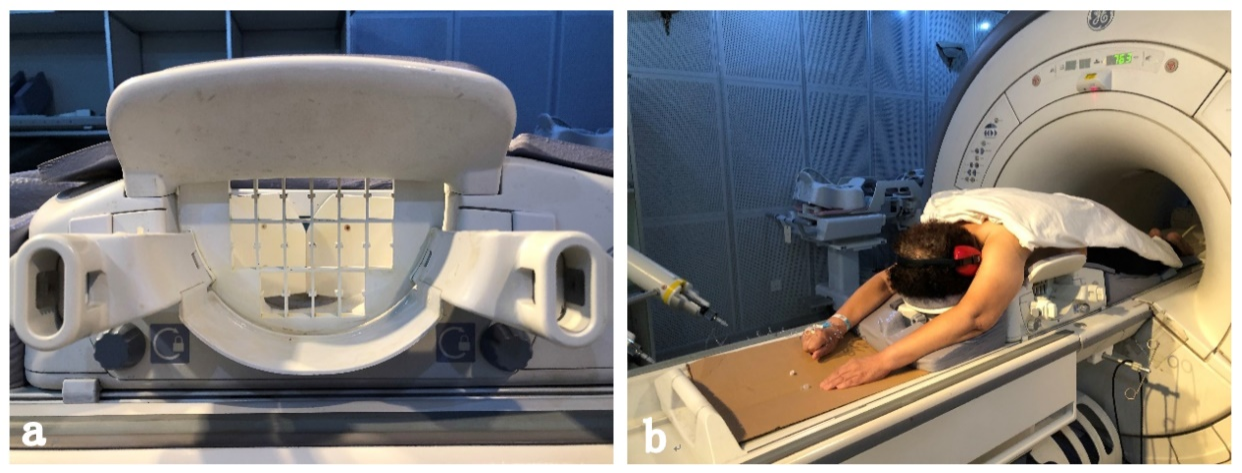
Preparations are significant for successful MRI- guided breast intervention. The coil (a)which is designed for MRI-guided intervention. A Patient is positioned prone in the coil (b) for the examination of MRI, with a mild compression for the breast. A headphone connected to the machine is used to reduce the noise of the MRI room. The arms of the patient are positioned above the head to avoid wrap artifacts and some newer coil techniques allow the arms to be positioned at the sides.

To improve the efficiency, the intervention equipment, such as the configuration of the coil, and patient body habitus which can influence the biopsy approach, should be prepared before a patient enters the MR imaging suite (**Figure 2**). Besides, the placement of grids which determine the access of the intervention should be carefully prepared. Gentle breast fixation is used to avoid the lack of enhancement of a lesion, and because of the compression of the breast, it is necessary to carefully evaluate the parenchyma surrounding the enhancing target for reliable anatomical landmarks 18.

For patients, receiving MRI-guided interventions are usually regarded as an intense, even scary experience, so before written informed consent, it's vitally important for surgeons and radiologists to communicate with their patients to discuss the reasons for the requested intervention, and the possible events that might occur during the procedure. Goals, benefits, the expected time the procedure might take and potential risks, and the possibility of cancellation should be explained before the operation. A study reported that listening to guided meditation lowered biopsy pain during the biopsy and that meditation and music reduced patient anxiety and fatigue 37. The patient collaboration is the key to the success of the procedure because the patient should remain still during the procedure, so the comfort of the patient during this period is essential to avoid lengthening the procedure time and compromising targeting accuracy. The patient should also be warned about the concept of non-visualization and the possibility, depending on lesion location within the breast, that there will be no safe access for sampling or localization 16,38. Of course, radiologists and technologists mush check patients who have any contraindications to MRI and/or gadolinium-based contrast administration when performing any contrast-enhanced breast MRI, during the previous breast MRI examination or the current MRI-guided intervention 39.

Successful MRI-guided intervention is truly a teamwork effort from patients to the surgeons, radiologists, MRI technologists, and nurses.

### Lesion Identification and Targeting

Radiologists should confirm triplane localizer images before the day of the intervention. Precontrast and postcontrast T1-weighted fat-saturated gradient echo sequences are performed. A fiducial marker (visible on T1- weighted sequences) is usually within the grid to facilitate lesion localization. The pre-contrast sequence should be reviewed to verify the approximate location of the lesion and the quality of fat saturation, the grid and the fiducial marker. Although some coils have standard locations where the fiducial is placed, put the fiducial in a grid square that is relatively close to the approximate location of the lesion but unlikely to be the exact entry location is recommended 40. And it is important that the imaging protocol should minimize image acquisition time while maintaining lesion visualization, because there is a short period following the administration of the intravenous contrast during which the area of interest can be visualized 41,42.

The coordinates of the chosen biopsy site can be subsequently calculated from computer software [eg, commercially available computer-aided diagnosis (CAD) tools] or manual localization method, and both methods of localization are usually used together. CAD system can assist radiologists in the analysis of breast MRI to reduce the interpretation time of analyzing breast MRI data. Albert Gubern-Mérida et al. 43 propose a novel CAD system to detect breast lesions in dynamic contrast-enhanced MRI automatically. As for the manual localization method, landmarks including: vessels, biopsy clips, and other foci of background parenchymal enhancement can be used to help localize the target 12.

**Figure 3:**
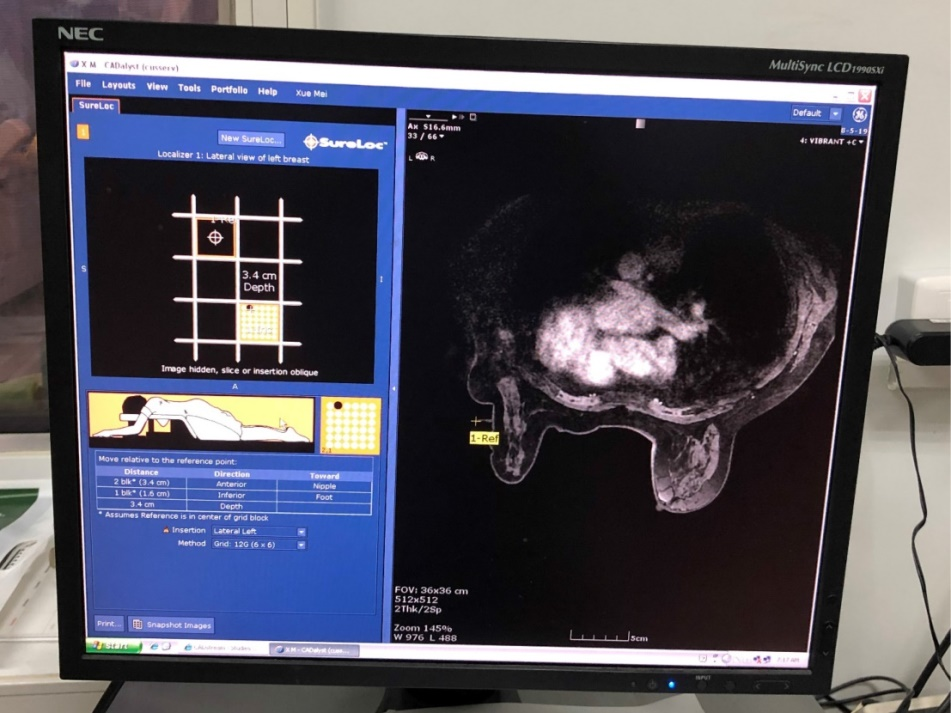
Computer-aided targeting in a 47-year-old woman with right breast lesion which is identified in the computer-aided detection (CAD) system. The CAD system provides a graphic depicting the optimal entry point for the needle (yellow crosshairs), and it also calculates coordinates of the lesion towards the nipple and foot. Especially, the lesion depth is important for MRI-guided intervention.

To target the lesion, the grid-guidance system and fiducial marker are adopted to determine the lesion coordinates (the x, y and z axes). Also, the CAD software can be used to calculate the skin entry site, select the correct needle insertion depth, and determine the optimal needle type, which can short the procedural time (**Figure 3**). Radiologists can obtain depth (z axes) of the lesion by counting the number of images from the skin surface where the grid is visualized to the targeted lesion and multiplying that number by the slice thickness. Remember that the skin surface is designated as a slice where the grid indentations on the skin are best visualized. Besides, a postcontrast axial, fat-saturated T1 sequence can also be used to measure lesion depth from the overlying skin. Alternatively, built-in scanner software can be used to records the depth coordinates for the lesion and the fiducial 2,18.

### Aseptic Technique before the Intervention

Before the intervention, technologists should clean and anesthetize the entry site using the sterile technique 44. Pain associated with local anesthetic administration may be improved by slow injection and buffering with sodium bicarbonate (8.4%), oral pain medication administered to the patient before the procedure, or i.v. sedation 23. For superficial anesthesia, lidocaine buffered in sodium bicarbonate may be used to reduce the initial stinging sensation of the lidocaine injection. And alternative drugs have to be used if the patient is allergic to lidocaine 45.

## Specific applications of MRI-guided detection and therapeutic methods in human breast

### MRI-guided Biopsy

#### Biopsy system

When performing the MRI-guided biopsy, it is significant for radiologists and MRI technologists to be very familiar with the components of the coaxial biopsy system and follow a correct way.

Vacuum-assisted large-gauge systems are recommended for biopsy, due to reasons of the challenges in precise targeting, the difficulties of MRI-guided biopsy and confirming lesion retrieval. It is crucial to select suitable commercial devices which are available from 8-12 G sizes, in order to optimize tissue sampling when targeting enhancing lesions that are in challenging positions 46.

**Figure 4:**
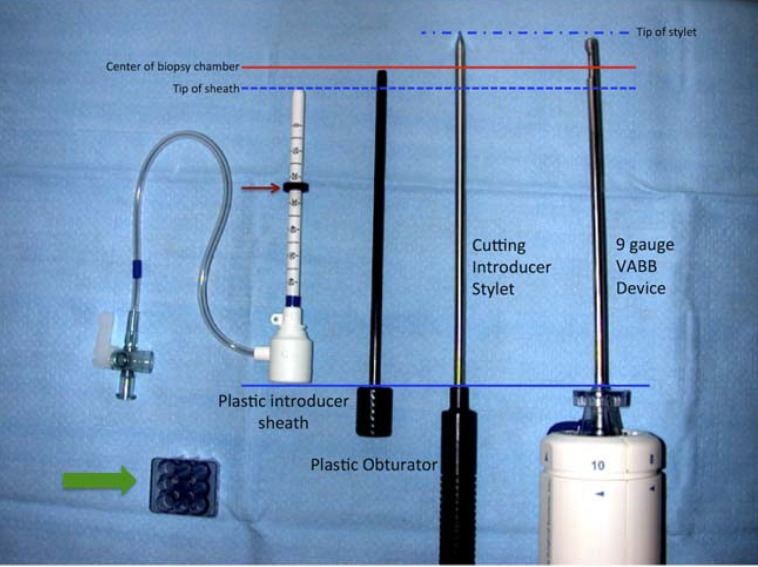
Components of an MRI-compatible coaxial biopsy system. The plastic introducer sheath is placed into the tunneled needle guide (wide green arrow) and advanced to the appropriate depth within the breast using a cutting introducer stylet. A rubber stopper (narrow red arrow) is used to set the appropriate depth. In this system, the plastic obturator is used to indicate the center of the biopsy chamber of the vacuum-assisted breast biopsy (VABB) device (solid red line) on prebiopsy images. Reproduced with permission. Modified and reprinted from McGrath AL, Price ER, Eby PR, Rahbar H. MRI-guided breast interventions. J Magn Reson Imaging. 2017. 46(3): 631-645. Copyright © 2017 International Society for Magnetic Resonance in Medicine.

The biopsy system generally contains: (1) an outer plastic introducer trocar/sheath with numbers indicating insertion depth and with an adjustable depth stopper, (2) a cube-shaped plastic tunnel needle guide that fits into the biopsy grid, (3) a cutting introducer stylet that helps to advance the plastic introducer trocar/sheath to the appropriate depth, and (4) a plastic obturator that is used to indicate the center of the biopsy chamber of the vacuum-assisted breast biopsy device on prebiopsy images (**Figure 4**) 18,47.

Adequacy of sampling is significant during the biopsy. It is typically performed with 12 samples (usually 1 sample per o'clock position), and additional samples may be obtained if the targeting is offset 2,48. The biopsy device usually allows manual rotation of the biopsy needle to sample the lesion preferentially. During the procedure, operators must always remember that the clockface is relative to them and it may be easier to direct sampling towards the body part (e.g. head, nipple, feet, or chest wall) or landmark (e.g. floor or ceiling). When completing the sample, lavage and aspiration of the biopsy cavity are performed to minimize the risk of post-biopsy hematoma and reduce imaging artifacts at the biopsy site. Tissue samples are placed in 4% formaldehyde and sent for histopathology 6,49.

**Figure 5:**
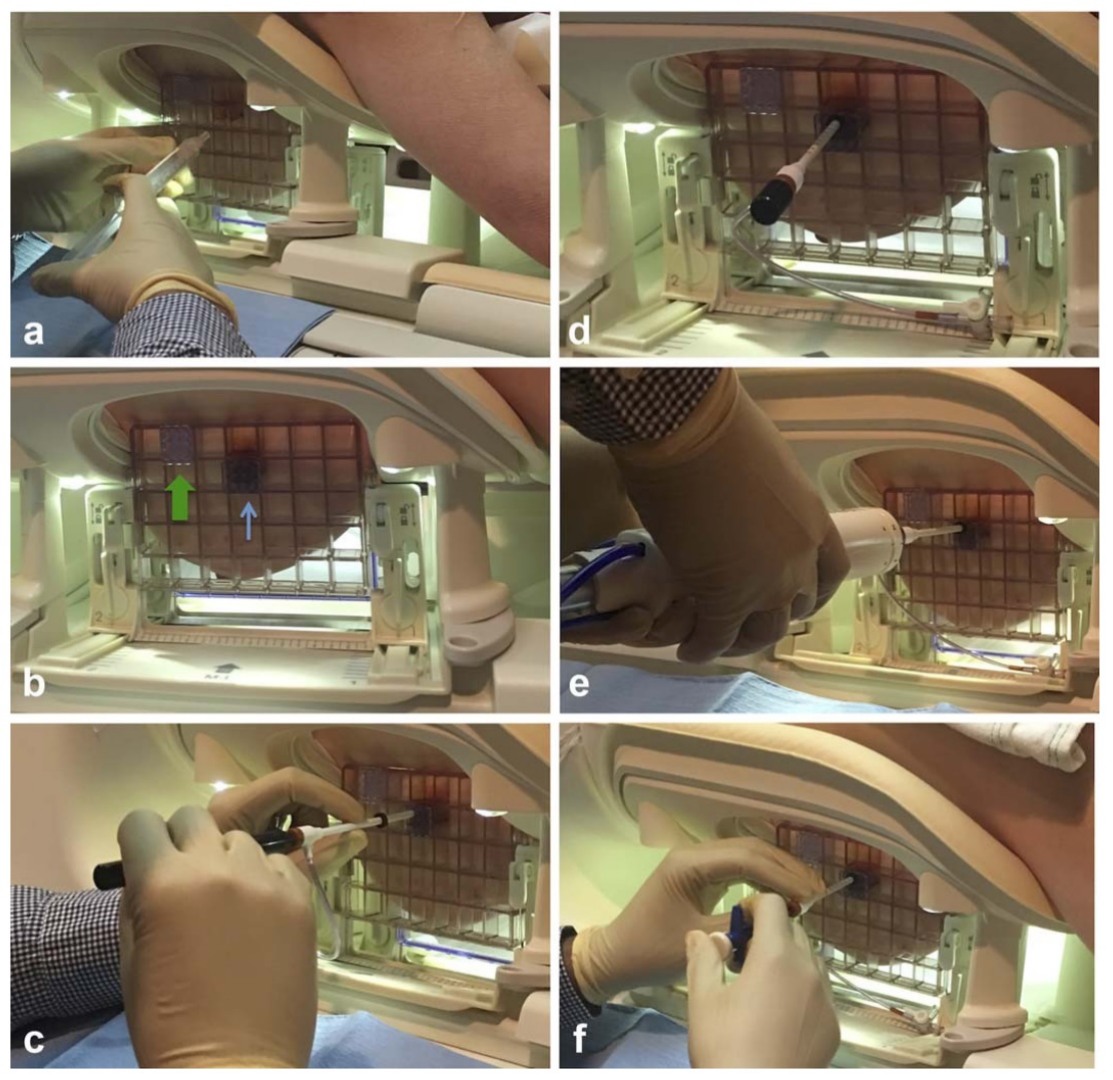
Example of typical steps involved when performing an MRI-guided breast biopsy. After determining the appropriate grid space for the skin entry site, the skin is cleansed and anesthetized (A), and local anesthetic is administered into the deeper breast tissues. The tunneled needle guide is then placed in the grid space where the biopsy (thin blue arrow) noting location relative to the fiducial (wide green arrow) (B). Using a “twist and push” motion, the plastic introducer sheath is placed into the breast through the appropriate hole in the tunneled needle guide with the cutting introducer stylet inside the sheath until the sheath reaches the appropriate depth as indicated by the rubber stopper (C). The cutting introducer stylet is subsequently replaced with a plastic obturator, and the patient is placed back in the bore of the MRI scanner so that a confirmatory T1-weighted sequence can be performed to confirm the appropriate location (D). The table is then brought out of the bore and the plastic obturator is replaced with the vacuum-assisted breast biopsy (VABB) device and multiple cores are obtained (E). After the sampling is complete, the VABB device is removed and a biopsy marker clip is deployed through the sheath (F). Modified and reprinted from McGrath AL, Price ER, Eby PR, Rahbar H. MRI-guided breast interventions. J Magn Reson Imaging. 2017. 46(3): 631-645. Copyright © 2017 International Society for Magnetic Resonance in Medicine.

Lastly, MRI-compatible metallic clip, visible on MR, mammography and ultrasound, is deployed at the biopsy site upon removal of the biopsy device. Metallic clips are usually used to mark the lesion in case there is a need for additional needle biopsy or excision following the pathology report. For cancer patients who choose to have breast-conserving surgery, MRI-guided clip placement of a broad area of ​​known disease or adjacent satellites before neoadjuvant therapy facilitates preoperative localization. A rapidly developing hematoma may interfere with further sampling, so the metallic clip should be placed, and the hematoma should be managed immediately. A post-biopsy T1-weighted image is then obtained to ensure the lesion is accurately targeted and it also allows the radiologist to confirm the placement of the clip. Whether to confirm the position of the clip with an MRI sequence has no consensus. However, a two-view mammogram is recommended which can help to confirm clip mark placement due to the postsurgical blood products and air can also obscure the susceptibility artifact. Because the compression of the breast may make a distortion, the location of the marker is not necessarily a reliable assessment of the sampling accuracy 2,16. Any clip displacement should be estimated and documented under the comparison of mammography and MRI. Li et al. 50 observed that the distance between the metallic clip and the lesion was not a reliable indicator of biopsy accuracy (**Figure 5**).

#### Challenging Clinical Situations

Some clinical situations may be somewhat challenging for technologists to have MRI guided biopsy. As for superficial lesions, a “half window” of sampling aperture is usually used in many systems to minimize skin damage and a blunt-tipped needle can be used to avoid skin damage 16. If the grid obscures the optimal entry site, causing the needle entry to be offset to the underlying target, the directional sampling method may also be needed 2. In some institutions, the freehand method is used for MRI-guided breast biopsy, which is different from other facilities that use a grid and contrast enhancement prior to needle placement. This method does not need specialized grid devices, and it allows for lesion localization throughout the breast, including lesions near the chest wall, in the axillary region and markedly posterior lesions 42,47. Lee JM et al. 51 reported an 8% to 13% cancellation rate of the procedure. Non-visualization of the suspicious finding may be due to change in tissue enhancement as the patient is in a different phase of her period or may be related to compression of breast tissue with decreased inflow of contrast material 52.

When multiple lesions are required to be biopsied, more than one technologist or radiologist may be helpful, and the biopsy supplies or equipment should be set up in advance. The biopsy devices should be available to be quickly set up once the targets are visualized to take advantage of the enhancement time and to prevent errors caused by patient movement.

Biopsy for women with breast implants needs to have informed consent for the risk of implant rupture. Dedicated technologists may provide invaluable help in displacing the implant to minimize the risk of implant rupture and ensure the adequacy of sampling 2,16.

### MRI-guided Localization

#### Techniques of MRI-guided Localization

In addition to MRI-guided biopsy, radiologists and breast surgeons should be familiar with MRI-guided localization. MRI-guided localization allows guidance of surgeons to the lesion to obtain both a complete removal (clear margins) and a good cosmetic result 53. Preoperative localization is often performed when MRI-guided biopsy is not feasible due to technical factors or patient's preference, such as the target lesion that is not accessible with MRI-guided biopsy 4,54. This approach is often needed by breast surgeons to help them perform breast-conserving surgery when the lesions are only visible with MRI, and to guide surgical re-excision of residual disease in cases of positive margins. Besides, MRI-guided localization is also used to demarcate lesions adjacent to benign enhancing structures, such as a scar or breast implants 24. And when neoadjuvant therapy is under consideration in order to mark the lesion site for re-evaluation or treatment planning, MRI-guided localization can also be performed 55.

When the coordinates of the target have been identified through computer software which is in the previous description, the skin entry site and optimal needle trajectory would be selected, and the needle guide and the needle are introduced through the grid hole overlying the skin 56. In the method of freehand technique, the needle trajectory should base on the desired surgical approach, and a full range of angled paths within the axial plane can be available as far as possible. The location of consequential and special structures should be avoided, such as implants. The skin entry site is marked with a pen after lesion identification 54,57.

After sterilization and anesthetization, a needle guide is used for accurate wire placement, and an MRI-compatible hook needle is inserted into the marked skin entry site and directed along with the planned needle approach to the appropriate location with the help of the grid-localization system. If a needle guide was used, an additional depth (typically 2 cm) should then be added to the calculated depth 2. The necessary steps of MRI-guided localization are almost the same as the process of MRI-guided biopsy, and MRI-guided localization allows for conservative surgical excision of a limited amount of tissue, yielding together an effective treatment and good aesthetic results 18,58-60. During the procedure of MRI-guided localization, dynamic real-time MR imaging can be performed to help visualize the placement of the needle and the release of the guidewire. Moreover, the radiologist can see and adjust the procedure of localization through a monitor that displays the MR images in the imaging room. The position of the needle can be immediately corrected if a shift of the breast tissue or a deviation of the needle is visible under the control of real-time MR imaging. The 1-cm marks on the shaft of the MR-compatible needle can be used to adjust the needle's position 61. Finally, when the position and the calculated depth are deemed satisfactory, the insertion of the needle is stopped, and the guide hook wire is released.

**Figure 6:**
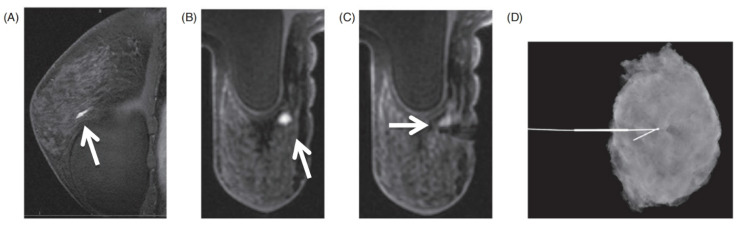
A 43‐year‐old female with a strong family history of breast cancer. A and B, Sagittal and axial T1‐weighted postcontrast images from screening MRI with an enhancing mass anterior to the left implant (arrows). There was no sonographic correlation. C, Axial T1‐weighted postcontrast MRI shows successful MRI‐NL (arrow). D, Specimen radiograph shows the wire in place after excisional biopsy. Pathology showed pseudoangiomatous stromal hyperplasia and fibroadenomatoid changes. Reproduced with permission. Modified and reprinted from Raj SD, Agrons MM, Woodtichartpreecha P, et al. MRI-guided needle localization: Indications, tips, tricks, and review of the literature. Breast J. 2019. 25(3): 479-483. Copyright © 2019 Wiley Periodicals, Inc.

The hook of the wire should be located beyond the target, which is analogous to that of mammographic-guided wire localizations. But due to that the compression of the breast is in the same direction as the wire placement approach in the MRI coil, MRI-guided wire localizations are more prone to postprocedural wire migration than mammography 18. Postprocedure two-view mammography should then be performed to confirm the position of the wire prior to surgery and to help the breast surgeon in determining the wire position (**Figure 6**) 62.

#### Challenging Clinical Situations

For multiple lesions and bilateral lesions, localization is usually performed of the more suspicious lesion or the smaller lesion, followed by localization of the remaining lesion. The localization of superficial target lesions is often performed using a particular marker, such as vitamin E, over the skin because there is not enough tissue depth to adequately secure a localization wire in place 24. For lesion close to the nipple, where the nipple may be difficult to stabilize, the needle may be placed adjacent to the lesion instead of through the lesion 56.

Nonvisualization may occur when radiologists perform the MRI-guided intervention at the rate of 12-13% 63,64. The majority of these lesions likely represent fluctuating physiologic BPE, but the absence of enhancement does not exclude malignancy. ACR Practice Parameters recommend “short-term follow-up,” and 3-6 months MRI follow-up is often suggested 18. MRI-guided intervention should be performed if the lesion is reappeared on follow-up, especially if the lesion is enlarged or shows malignant signs.

## The combination of MRI-guided methods and other technologies in human breast

### Considerations after the Operation

Once the procedure has been finished, the radiologist initiated manual hemostasis within the MRI suite. The puncture location of the breast should be sterilized again, and manual compression, usually for 5 min, as well as cooling, is applied followed by a compressive dressing. Then the patient is moved out of the magnet and kept warm with clothes. It is generally considered that half an hour of monitoring after the surgery is usually sufficient, and the radiologist should provide the patient with postprocedural care instructions 41,65. Besides, efforts also should be made to decrease patients' distress and anxiety, which is important general aspects for patients and the quality of the patient-doctor relationship 66.

### New Techniques for Lesion Localization and MRI-Guided Breast Interventions

Currently, techniques available for MRI-guided intervention are developing quickly, and the market for alternative methods is increasing. With the increase of patients who is eligible for breast MRI, faster, cheaper and accurate MRI techniques will also be needed.

In order to make the MRI scan faster, many are investigating “ultrafast” techniques that complete postcontrast imaging within 30 seconds of contrast injection 67,68. Breast MRI offers the potential for the measurement of fibroglandular tissue volume to assess breast density, and Gokhan E et al. 69 developed a fast, computerized algorithm for volumetric breast segmentation, suitable for multi-center data, employing 3D bias-corrected fuzzy c-means clustering and morphological operations to make the assessment more accurate. Moreover, statistical analysis shows that the segmentation algorithm performs well when compared with manually corrected segmentation in a UK multi-center study of MRI screening with eighty-two women scanned.

Apart from contrast-based MRI protocols, non-contrast MRI techniques, such as diffusion-weighted imaging (DWI), are developing. As a functional MRI technique, DWI has the benefit of not requiring intravenous gadolinium, takes less time and is of lower cost than a full protocol traditional contrast-enhanced MRI. Besides, lesion detection and characterization, and the evaluation of neoadjuvant treatment response of DWI are also promising applications 70-72. Nicole B et al. 49 evaluated the feasibility of DWI for lesion targeting in MRI-guided biopsies, and concluded that the targeted lesion requires the characteristics of a mass-like lesion, substantial diffusion restriction, and a minimum size of approximately 1 cm.

Samuel BP et al. 73 developed an MR-compatible needle intervention robotic system, which can approach the tumor from the side with a bendable needle. This new design solves the space limitations imposed by the MR gantry and breast coils. The position accuracy is increased with the system's image-guided feedback software.

With the preoperative supine MRI, Barth RJ Jr et al. 74 outline the tumor edges on consecutive images and create a bra-like plastic form (the Breast Cancer Locator [BCL]) that is manufactured for each patient using a 3D printer. The BCL can transmit both surface and intraparenchymal information to locate the tumor precisely in the OR and can help the surgeon to perform the breast-conserving surgery with the goal of removing 1 cm of normal-feeling tissue around the palpable tumor.

In addition to needle/wire localization aided guidance, in recent years, more and more new technologies are born that allow for localization up to several days before surgery, like carbon marking, radioactive technetium-99m (99mTc) localization for lesion and sentinel lymph node, radioactive iodine-125 (125I) seeds localization, magnetic seed localization and SAVI SCOUT radar localization. These new technologies can relieve the pressures of the number of same-day requests for diagnostic localization or biopsy of breast imaging centers, as well as increase patients' convenience and comfort. However, these newer techniques cannot be placed under MRI guidance, and to date only wire localizations can be performed under MRI guidance 75-81.

## Main advantages of MRI-guided detection and therapeutic methods in human breast

MRI remains the most powerful tool for detecting clinically suspected lesions which are mammographically and sonographically occult. MRI-guided biopsy or localization is essential in the management of suspicious breast lesions detected by screening or during the assessment of clinical abnormalities. It is a safe and cost-effective procedure allowing for an accurate diagnosis, pivotal for adequate decision-making, including, when indicated, treatment planning 82,83. MRI has a sharp delineation of breast lesions, which is a right choice of the imaging modality to help the surgeon choose surgical approaches between BCS and mastectomy. Before performing BCS, the surgeon must use the MR images to confirm multicentric or single tumor of the breast, and review the size and location of the tumor combining with other equipment to ensure the success of the operation. Breast surgeon needs to minimize the amount of tissue excised and to ensure that clear surgical margins are as much as possible achieved before making the first incision^11^, ^84^, ^85^. Besides, MRI-guided biopsy is also used to prevent unnecessary surgery, associated morbidity and costs for equivocal findings on imaging with final non-malignant histopathology 86.

For MRI-guided intervention, complications are infrequent and mild 2. Major complications during or after vacuum biopsy include infection, hematoma formation, pneumothorax and skin damage, with the complication rates ranging from 0 to 6% 65,87-90. And these risks can be minimized with careful biopsy planning and selection of an appropriate biopsy device. The risk of mechanical displacement of malignant cells along the biopsy tract can very rarely occur and is referred to as neoplastic seeding. Brenner RJ et al. 91 reported that the risk of this event was to be 2 of 1644 biopsies, and Santiago L et al. 92 reported the incidence was to be 8 of 4010 biopsies.

For MRI-guided localizations, complication rates reported in the literature are 0~3.3% 10,24,54,57,59,61,93. Complications consist of wire dislocations without relevance to the planned surgical procedures, bleeding out of the puncture channel, and hematoma, etc. Kathrin Barbara Krug et al. 10 performed 347 patients with MR-guided wire localization and reported that the complication rate is 3.3% and it was not influenced by patient age, clinical referrer, the indication of the wire localization, or histology of the operative specimen.

## Main disadvantages of MRI-guided detection and therapeutic methods in human breast

The MRI-guided intervention is safe and accurate in specialized centers and relatively fast, but longer and more costly if compared to other imaging-guided procedures.

For the biopsy, the average time spent in the MRI is from 20 to 90 minutes, and this is increased by 30 to 50% when two sites are biopsied 65,94-96. Mitra Noroozian et al. 41 reported that the duration of the procedure depends on the number of images and checks in the tunnel, whether or not an assistant is present and proximity to the nipple, and it is independent of patient age, size of the breast and type or size of enhancement. For needle localizations, the entire operation lasts for about 40 minutes 6,10,61,73,93,97. Krug KB et al. 10 warrant that it is beneficial to reduce the time of the interventional procedure to increase the patient's convenience during the intervention to avoid target lesion shadows due to contrast washout, to avoid inaccurate definition of target lesions due to motion artifacts, and to improve the ability of the MRI scanner.

## Perspectives and Conclusion

Correlation between radiology and pathology is the final step after MRI-guided interventions, and it has unique challenges between benign and malignant lesions. For cases that have been assessed as possibly missed or discordant, a repeat biopsy or surgical excision is recommended. MRI-guided core biopsy malignancy rates varying between 16-40.91% have been reported 4,32,41,98-101. For benign-concordant lesions, a diagnostic follow-up MRI after 6 months of MRI-guided interventions is recommended due to the risk of false-negative results 18,50. And as for malignancy, doctors need to choose open surgical methods according to the condition of the patient.

MRI provides very high sensitivity for the detection of breast tumors which can't be seen with traditional breast examination equipment, based on anatomical and functional information, and it provides important guidance for the diagnosis and treatment of occult breast carcinomas. As for the subsequent treatment, preoperative MRI-guided intervention plays a vital role in the choice between BCS and mastectomy. Whether benign or malignant concordant lesions, preoperative MRI-guided biopsy provides necessary information for doctors and patients with a strong willingness for breast-conserving. Also, preoperative MRI-guided wire localization successfully and virtually ensures the performance of diagnostic excisions of suspicious breast lesions that can only be seen in MRI, and it also ensures the possibility of breast-sparing resections for the patients with single cancer of breasts.

Although MRI-guided intervention is more complicated, take more time and are more costly than other lesion image-aided localization technique. It offers the benefits high sensitivity rate for suspected lesions of MRI while minimizing false-positive findings and improving specificity by tissue sampling. What's more, it yields high technical and clinical success rates, low complication rates, and only minor forms of complication.

This work was supported by the Natural Science Foundation of China (No. 81872160), the Beijing Municipal Natural Science Foundation (Key Project) (No. 19G10077), the Beijing Municipal Natural Science Foundation (No. 7204293), the Special Research Fund for Central Universities, Peking Union Medical College (No. 3332019053), the Beijing Hope Run Special Fund of Cancer Foundation of China (No. LC2019B03), the Beijing Hope Run Special Fund of Cancer Foundation of China (No.LC2019L07), the CAMS Initiative for Innovative Medicine Foundation (No.2017-I2M-3-020).
